# Replication Protein A (RPA) Hampers the Processive Action of APOBEC3G Cytosine Deaminase on Single-Stranded DNA

**DOI:** 10.1371/journal.pone.0024848

**Published:** 2011-09-15

**Authors:** Artem G. Lada, Irina S.-R. Waisertreiger, Corinn E. Grabow, Aishwarya Prakash, Gloria E. O. Borgstahl, Igor B. Rogozin, Youri I. Pavlov

**Affiliations:** 1 Eppley Institute for Research in Cancer and Allied Diseases, University of Nebraska Medical Center, Omaha, Nebraska, United States of America; 2 National Center for Biotechnology Information, National Library of Medicine, National Institutes of Health, Bethesda, Maryland, United States of America; 3 Institute of Cytology and Genetics, Novosibirsk, Russia; St. Georges University of London, United Kingdom

## Abstract

**Background:**

Editing deaminases have a pivotal role in cellular physiology. A notable member of this superfamily, APOBEC3G (A3G), restricts retroviruses, and Activation Induced Deaminase (AID) generates antibody diversity by localized deamination of cytosines in DNA. Unconstrained deaminase activity can cause genome-wide mutagenesis and cancer. The mechanisms that protect the genomic DNA from the undesired action of deaminases are unknown. Using the *in vitro* deamination assays and expression of A3G in yeast, we show that replication protein A (RPA), the eukaryotic single-stranded DNA (ssDNA) binding protein, severely inhibits the deamination activity and processivity of A3G.

**Principal Findings/Methodology:**

We found that mutations induced by A3G in the yeast genomic reporter are changes of a single nucleotide. This is unexpected because of the known property of A3G to catalyze multiple deaminations upon one substrate encounter event *in vitro*. The addition of recombinant RPA to the oligonucleotide deamination assay severely inhibited A3G activity. Additionally, we reveal the inverse correlation between RPA concentration and the number of deaminations induced by A3G *in vitro* on long ssDNA regions. This resembles the “hit and run” single base substitution events observed in yeast.

**Significance:**

Our data suggest that RPA is a plausible antimutator factor limiting the activity and processivity of editing deaminases in the model yeast system. Because of the similar antagonism of yeast RPA and human RPA with A3G *in vitro*, we propose that RPA plays a role in the protection of the human genome cell from A3G and other deaminases when they are inadvertently diverged from their natural targets. We propose a model where RPA serves as one of the guardians of the genome that protects ssDNA from the destructive processive activity of deaminases by non-specific steric hindrance.

## Introduction

Deaminases of the AID/APOBEC superfamily play amazingly diverse roles in vertebrates [1]. APOBEC1 works in lipid metabolism by editing apolipoprotein B mRNA [1], [2]. AID is involved in immunoglobulin (Ig) diversification by initiating somatic hypermutation (SHM) and class-switch recombination (CSR) [3]. Members of the APOBEC3 subfamily restrict retroviruses and retrotransposons and have been implicated in the clearance of foreign DNA from human cells [1], [2], [4], [5]. PmCDA1 is involved in immunity in jawless vertebrates [6]. AID/APOBEC enzymes convert cytosines to uracils in their target nucleic acids and therefore are inherent mutators [2], [7] and cause single-stranded DNA breaks [8]. Improper targeting of deaminases could lead to point mutations and translocations, and ultimately to cancer [9]. Tight regulation of the activity of AID/APOBECs is vitally important for the prevention of genome instability. In agreement with the mutator properties of these enzymes, the expression of deaminases is mutagenic in heterologous hosts, such as bacteria and yeast ([1], [2], [10] and references therein). To gain insight into the mechanisms of genome protection from deaminase-dependent mutagenesis, we studied A3G-induced mutagenesis in live yeast cells and on the DNA of the same reporter *in vitro* using purified recombinant proteins. Analysis of the data obtained revealed striking differences between these two systems. A3G was non-processive *in vivo* but processive *in vitro*. In searching for the factors that suppress the processivity *in vivo*, we found that RPA inhibits both DNA deaminase activity and processivity of the A3G. Our data demonstrate that RPA may protect genomic DNA from the destructive activity of editing deaminases.

## Results

**Figure 1:**
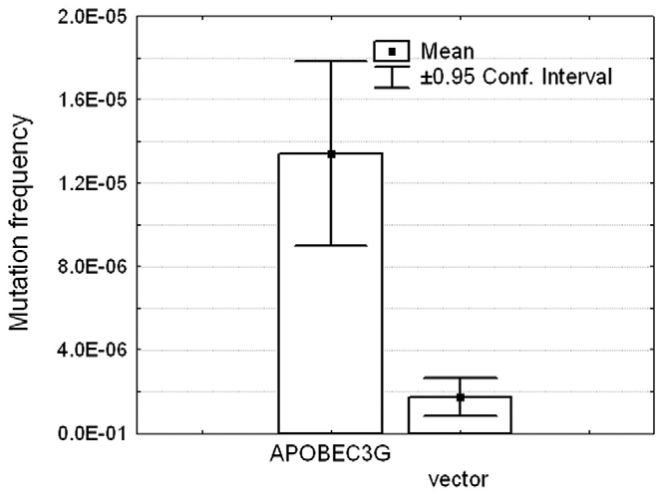
A3G is mutagenic in yeast. The frequency of 5-FOA-resistant colonies induced in the LAN-200 yeast strain carrying an A3G expression plasmid or vector alone is shown. One-way ANOVA F-test = 29.99, *p* = 0.00002.

**Figure 2:**
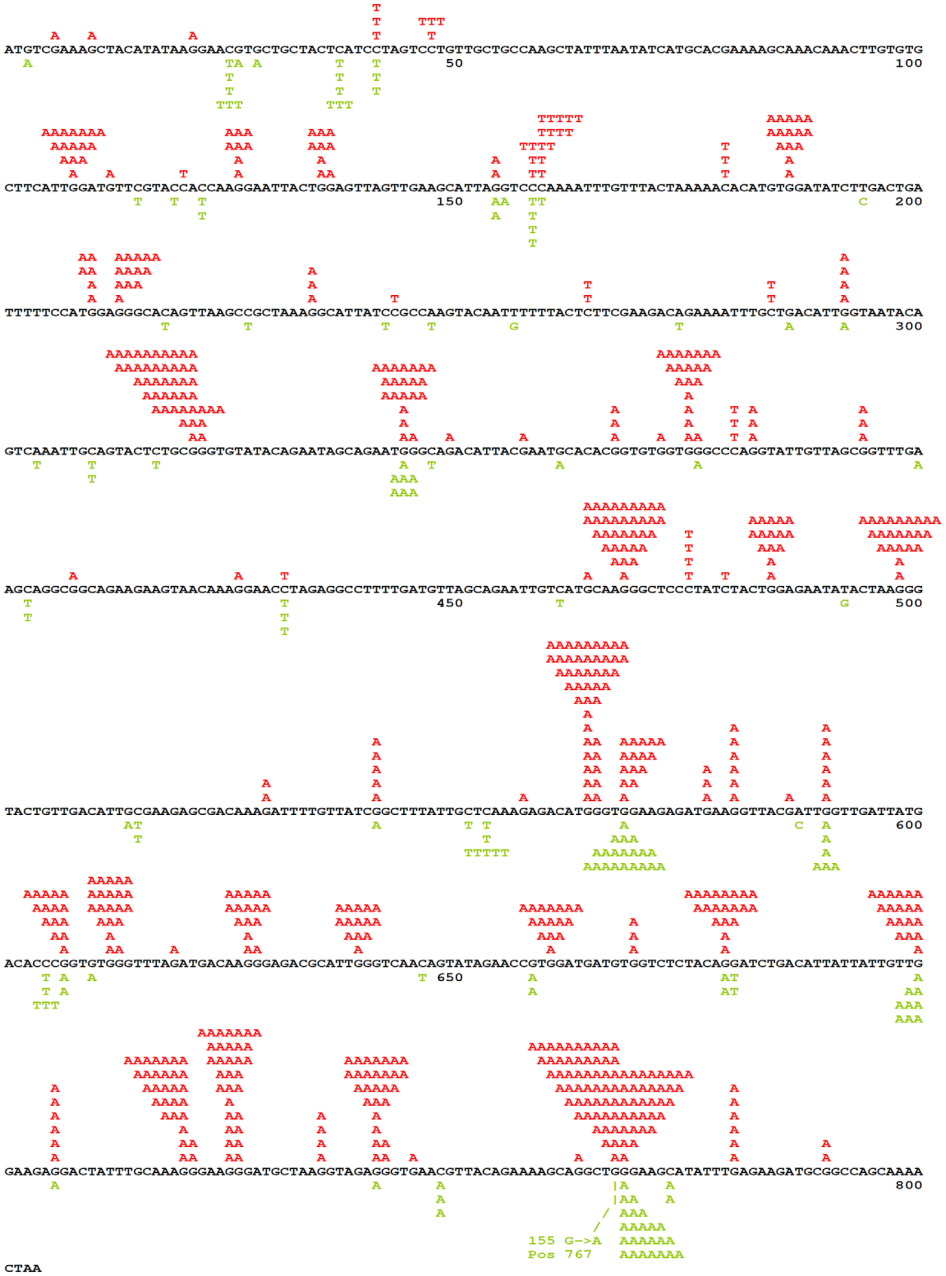
Spectra of mutations induced in the *URA3* gene *in vivo* and *in vitro*. Red letters over the *URA3* sequence indicate mutations found *in vitro* in the gapped substrate assay. Multiple substitutions found in *ura3* mutants induced by A3G in the gapped substrate in several experiments were pooled together. Green letters below the *URA3* sequence are the mutations induced by the expression of A3G in the LAN-200 yeast strain. C to T substitutions result from the deamination of the non-coding DNA strand, whereas G to A substitutions are the consequence of the coding strand deaminations. Most of *ura3* mutants obtained in yeast contained single base substitutions in the *URA3* open reading frame. However, we found two clones possessing two substitutions each: C159T (silent) and G767A (nonsense) in one clone, and G741A (silent) and G767A (nonsense) in the other clone. In addition, one clone contained duplication of CAGACA at position 347 (there is CCC motif on the opposite strand just before the duplicated sequence).

**Figure 3:**
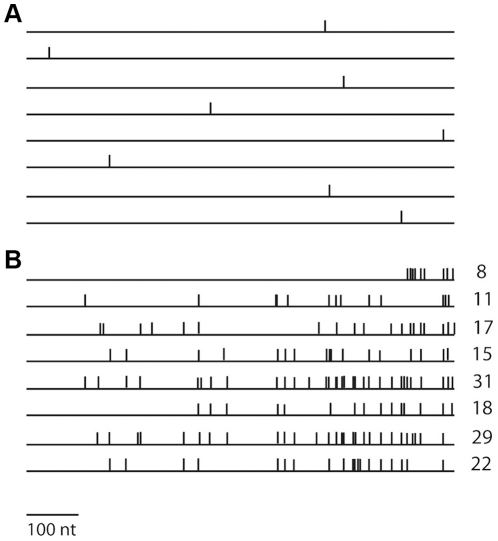
Comparison of mutations induced by A3G *in vitro* and *in vivo*. Schematic representation of examples of mutant *ura3* alleles obtained upon A3G expression in yeast cells (a) and *in vitro* using gapped substrate (b). Horizontal lines represent the *URA3* gene ORF; vertical bars indicate the approximate positions of base substitutions. Eight independent mutant clones are shown in (a) and eight in (b). The number of base substitutions per clone is shown on the right (b).

In the first step of this study, we analyzed the mechanisms and parameters of the A3G action on genomic loci in yeast. We expressed human A3G in a *S. cerevisiae* strain defective for uracil DNA glycosylase (*ung1*). Ung1 initiates the base excision repair of uracil-containing DNA by removing the uracil moiety so the effects of cytosine deaminases are stronger when Ung1 activity is absent. A3G production in the *ung1^−^* strain leads to about an eight-fold increase in the frequency of forward mutations at the *URA3* locus (Fig. 1), as determined by the frequency of colonies resistant to the 5-fluoroorotic acid (5-FOA). The mutagenic effect of A3G production in yeast suggests that this enzyme is able to penetrate the nuclei of yeast cells and deaminate cytosines in the genomic loci. As expected from the cytosine deamination, sequencing analysis of the *URA3* gene from 311 independent mutant clones revealed that almost all mutations were C to T or G to A transitions. Most of the substitutions were observed in the CCC “hotspot motifs,” which is the characteristic feature of A3G both *in vivo* and *in vitro* (Fig. 2, green letters) [11], [12]. The vast majority of the sequenced clones contained a single base substitution in ∼800 bp of the *URA3* open reading frame (ORF) (Fig. 3a). Only two clones among the 311 analyzed contained two substitutions, both found in the CCC motifs (one clone: C159T (silent) and G767A (Trp to STOP); another clone: G741A (silent) and G767A (Trp to STOP)). According to Poisson statistics (*p*<1), the mutants with double substitutions result from independent hits of APOBEC3G. These double hits occurred most likely at different generations in yeast culture. We concluded that A3G is not processive *in vivo* in yeast.

**Figure 4:**
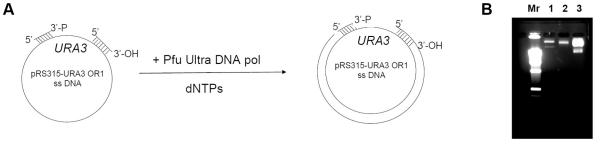
Construction of circular gapped DNA substrate for the *in vitro* deaminase assay. a. Scheme of substrate construction (See Materials and Methods for details). b. Agarose gel analysis of the reaction products. The gel was stained with ethidium bromide (EtBr). Mr – molecular weight marker, 1 – original ssDNA, 2 – ssDNA with two oligonucleotides annealed, and 3 – gapped substrate – product of Pfu Ultra reaction. Lanes 1 through 3 contain similar amounts of DNA by molarity. The gapped substrate band on lane 3 is much brighter than the ssDNA on lanes 1 and 2 because double-stranded DNA binds much more EtBr than the ssDNA of the same length due to intercalation of EtBr in the double helix. The lower bands on lanes 1 and 3 represent linear DNA species that arise due to damage to the circular ssDNA.

**Figure 5:**
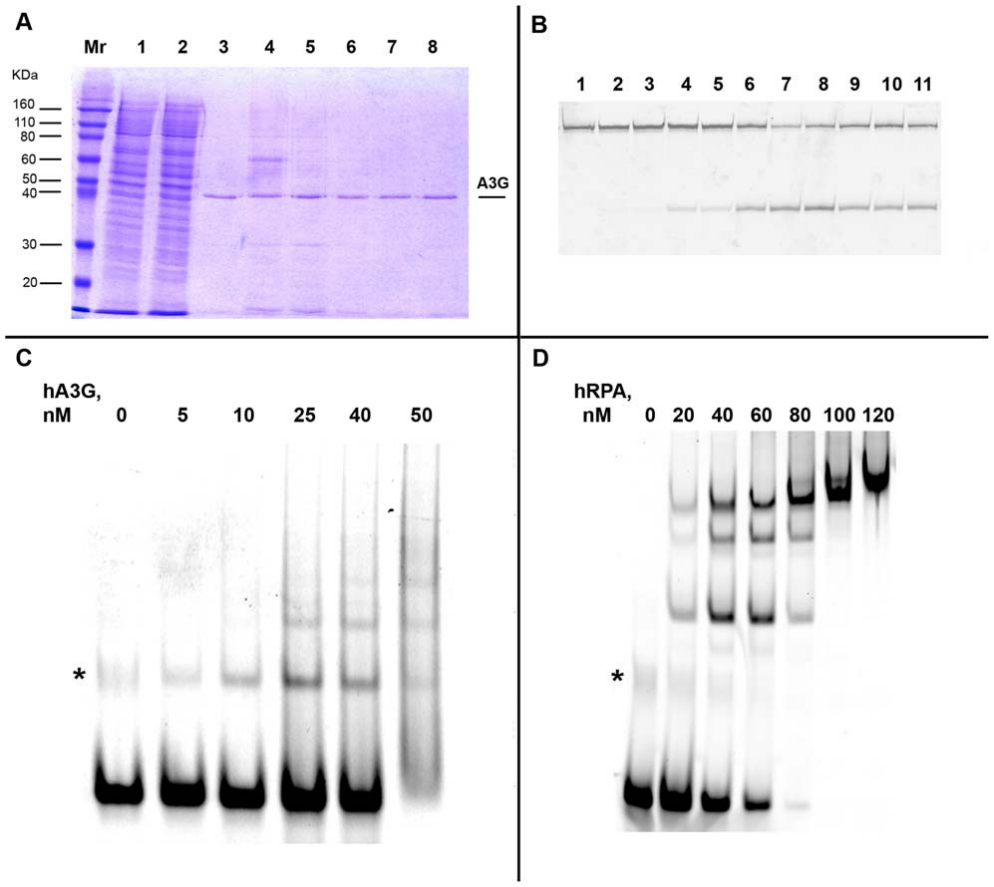
Purification and characterization of proteins used in the study. a. Coomassie-stained 12% SDS-PAGE gel of aliquotes from different steps of A3G purification from HEK293T cells. Mr – molecular weight marker, 1 – clarified lysate, 2 – flow through, 3–8 – different protein-containing fractions, eluted from the resin. b. Deaminase activity of purified A3G, detected in the oligonucleotide assay with uracil-DNA-glycosylase. In this assay, after deaminase converts cytosine to uracil, uracil-DNA-glycosilase removes uracil from the DNA, leading to formation of the AP site, which is further converted into strand break under conditions of high pH and temperature. 1 – UDG alone, 2 - A3G-expressing HEK293T lysate, 3 – clarified lysate, 4 – clarified lysate treated with RNAse, 5 – flow through, 6–11 – different fractions of purified protein. c and d. DNA-binding activity of purified A3G (c) and human RPA (d), detected by electromobility shift assay (EMSA). A3G from fraction 5 (see panel a) was used in this assay. The same oligonucleotide was used for both proteins (c). The band that corresponds to the free oligonucleotide folded to the secondary structure is indicated by the asterisk. Note that in (c) this non-specific band migrates similarly to the fastest A3G-shifted band.

It is established that A3G, as well as AID, is processive *in vitro*, which is generally defined by the ability to perform multiple deaminations upon one encounter with the DNA molecule (also see Discussion) [13], [14], [15]. In the next step of this study, we analyzed A3G activity *in vitro* using the same *URA3* reporter so we could directly compare these results with the in results obtained with the heterologous yeast system *in vivo*. Because AID/APOBEC proteins act only on ssDNA [1], [2], we used a modified *in vitro* deaminase assay with a gapped DNA substrate [13]. This substrate consists of a circular double-stranded DNA vector sequence and a single-stranded gap containing the *URA3* gene sequence. The substrate is treated with the deaminase and *ung^−^ pyrF^−^* bacteria is transformed by the reaction product. Individual transformants are selected and replica-plated on media with and without uracil to select *ura-* clones. This is possible because the yeast *URA3* is an ortholog of the *pyrF* gene of *E.coli* and compensates for the *pyrF* deficiency [16]. We developed a new DNA polymerization-based approach with the use of blocking phosphorylated oligonucleotide (see Materials and Methods and Fig. 4) to construct the circular gapped DNA substrate. We used two similar substrates that differ in the orientation of the *URA3* reporter and allowed us to examine targeting of the deaminase to the coding and non-coding strands of the *URA3* gene. Recombinant A3G was purified from the HEK293T cells transfected with the wild-type human A3G expression vector (Fig. 5a) [17]. The purified enzyme possessed robust DNA-binding (K_d_≈4.5×10^−8^ M) and deaminase (1.6 pmol µg^−1^ min^−1^) activity on short oligonucleotides (Fig. 5c, b, respectively). Incubation of the gapped substrates with the recombinant A3G resulted in a ∼20-fold increase in the frequency of mutants (4–8% Ura^−^ clones vs. 0.3% in the control). Multiple C to T or G to A (depending on the *URA3* orientation) transitions were found in the *URA3* coding sequences isolated from the mutant clones (Fig. 3b, Supporting Table 1). As many as 31 mutations per clone were found, with an average number of 17.3. A low fraction of mutant clones, along with the high numbers of base substitutions per clone obtained in this experiment, is indicative of enzyme processive action [13], [18], [19]. According to Poisson statistics (*p*<10^−7^ according to the χ^2^ test for the data of the experiment presented in the Table S1), all substitutions in virtually any single *ura-* clone result from one deaminase-substrate encounter event. The majority of base substitutions were found in the typical A3G hot motifs (Fig. 2, red letters). The average length of an A3G tract (which is defined as the distance between first and last substitutions) was 541 nucleotides, with 672 nucleotides being the maximum. Despite the fact that single, silent substitutions can not be detected in the selective system, we observed a highly significant correlation in mutable positions between the *in vivo* and *in vitro* spectra (Pearson linear correlation coefficient = 0.79, P<10^−6^). A striking difference in the proportion of multiple deaminations in our *in vitro* and *in vivo* experiments suggests that the high processivity of A3G is lost *in vivo* (Fig. 3).

**Figure 6:**
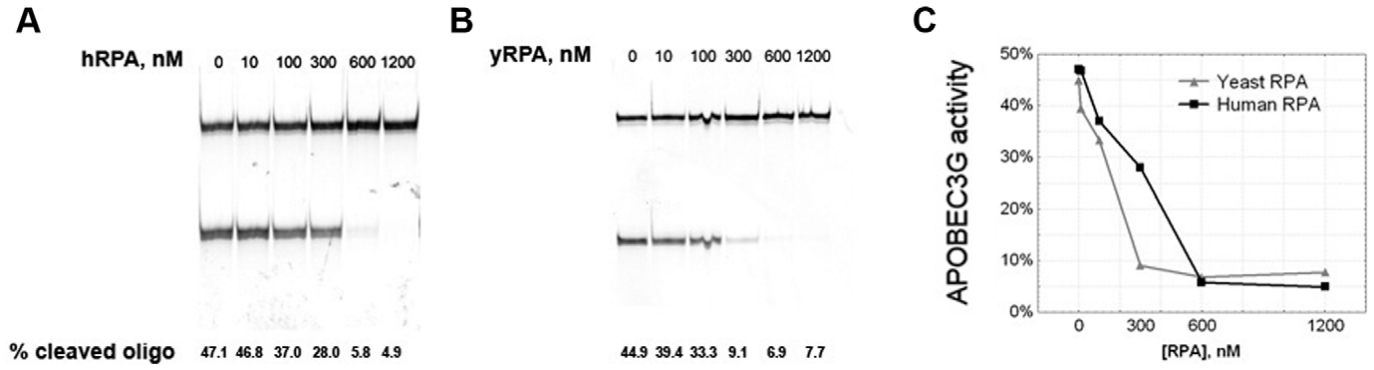
RPA inhibits deaminase activity of A3G *in vitro*. Oligonucleotide deamination assay (Materials and Methods and Fig. 5b) was performed with the addition of human (a) and yeast (b) RPA in various concentrations. Relative deaminase activity was calculated as a percentage of cleaved oligo. Activity data for A3G in the presence of human and yeast RPA were plotted as a function of RPA concentration (c).

The ssDNA in cells are always protected and covered by single-stranded DNA binding proteins, called Replication Protein A (RPA) in eukaryotes [20], [21]. We hypothesized that RPA protects the majority of genomic ssDNA from the activity of AID/APOBEC enzymes. We analyzed the effect of RPA on the deaminase activity of A3G using oligonucleotide deamination assay *in vitro* with pure proteins. Human RPA was purified as described [22], and yeast RPA was a gift of P. Burgers (Washington University). DNA-binding activity of both RPA preps was tested using electrophoretic mobility shift assay (Fig. 5d and data not shown). Both human (Fig. 6a) and yeast (Fig. 6b) RPA inhibited A3G activity in a concentration-dependent manner. A nearly complete inhibition of deamination reaction was achieved at the concentrations of RPA (∼300 nM for yRPA and ∼500 nM for hRPA), where all DNA in the reaction is expected to be covered with RPA, according to the published “footprints” of the corresponding proteins (Fig. 6c) [21]. This is similar to the data on the inhibitory effect of RPA on the activity of AID [23].

**Figure 7:**
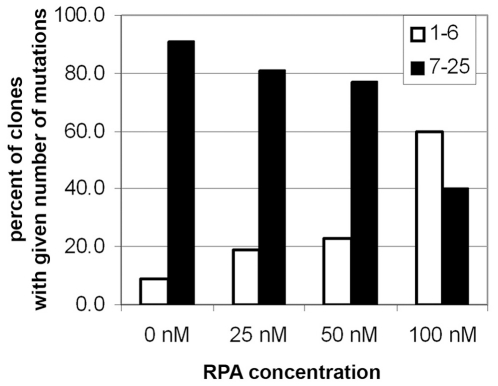
RPA inhibits A3G processivity *in vitro*. Proportions of *ura3* mutant clones with less (white bars) and more (black bars) than seven substitutions in the ORF are shown as a function of hRPA concentration. The observed differences between frequencies of clones with multiple mutations obtained in different experimental conditions are significant (P = 0.002 according to the χ^2^ test).

Next, we asked how RPA influences the deaminase activity of A3G on the gapped substrate containing a long stretch of ssDNA. The gapped substrate DNA was incubated with A3G in the presence of various concentrations of human RPA, and analysis of mutants was done as described previously. The addition of RPA (100 nM final concentration) to the reaction mix caused a modest decrease in the frequency of Ura^−^ clones from 7.6% to 3.7%, but, importantly, the frequency of clones with multiple mutations gradually decreased with the increase of RPA concentration (Fig. 7). The frequency of clones with less than seven substitutions increased from about 10% in the absence of RPA to 60% with the 100 nM RPA. We concluded that RPA inhibits not only deamination activity of A3G *per se* (Fig. 6), but also processivity of the enzyme (Fig. 7). This observation is consistent with the yeast *in vivo* data, where processivity of deaminase is almost absent.

**Figure 8:**
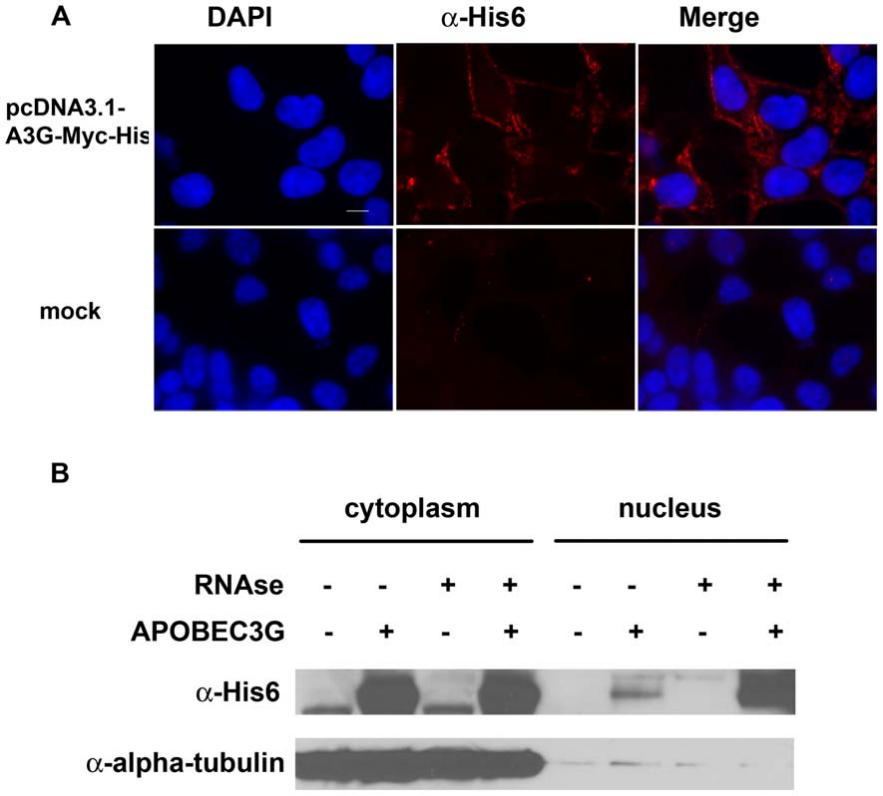
Nuclear-cytoplasmic distribution of A3G. (a) HEK293T cells transiently transfected with APOBEC3G-expressing plasmid were fixed and stained with DAPI to visualize nuclei and anti-His_6_ antibodies to visualize APOBEC3G. (b) Western-blot analysis of nuclear and cytoplasmic fractions of HEK293T cells overexpressing A3G. See Materials and Methods for details.

According to our data and previous reports, A3G is highly mutagenic in yeast, therefore it is able to penetrate the nuclei of cells [10], [24]. In mammalian cells, A3G is localized predominantly in the cytoplasm (for example, [25] and references therein), although a small but yet detectable fraction of the protein is found in the nucleus when A3G is expressed at the endogenous level, and an even higher level of A3G is found in the nucleus when the protein is overproduced [26]. A substantial fraction of cytoplasmic A3G is localized to P-bodies [27], [28]. We also found that overexpressed A3G in the cytoplasm of HEK293T cells is concentrated in the punctuate bodies (Fig. 8a). We did not detect A3G in the nuclei using immunofluorescence (Fig. 8a). However, fractionation of cytoplasmic and nuclear extracts of the same cells (see Materials and Methods) followed by Western blot analysis revealed that a small but detectable amount of A3G is found in the nuclear fraction (Fig. 8b). Moreover, RNAse treatment during the course of fractionation resulted in the increase of A3G in the nuclear fraction (Fig. 8b).

## Discussion

We have shown that A3G introduces predominantly single base substitutions per mutant in the yeast genomic locus, whereas it is highly processive *in vitro* on the same *URA3* gene reporter and produces clusters of mutations in each mutant (Fig. 3). A3G introduces multiple mutations both on its natural target, the viral cDNA, and in Ty retrotransposon cDNA in the yeast system [24], [29], [30]. Similar to A3G, AID is processive *in vitro* and induces multiple deaminations [13]. In this paper we define processivity in a broad sense, as the ability of the enzyme to catalyze multiple reactions per each substrate encounter, before moving to the other substrate molecule [15]. Tracts of mutations are present in the Ig genes that undergo somatic hypermutation, but the multiplicity of mutations is less than *in vitro* [31]. It is also possible that multiple substitutions in the immunoglobulin genes could partially result from consecutive selection of lymphocytes. Multiple substitutions are found in the oncogenes mistakenly targeted by the AID, but the number of substitutions per gene is usually less than in Ig genes [32], [33]. Comparison of the spectra of the mutations in the ultimate targets of deaminases to random genomic reporters clearly indicates that there is a mechanism protecting the majority of the genome from destruction by deaminases. Various structural proteins of chromatin, proteins involved in transcription [31], DNA replication and repair enzymes could potentially endow this protection. On the other hand, natural targets of deaminases *in vivo* should be found in a special microenvironment (or subcellular compartment) where certain protective components are missing or modified.

**Figure 9:**
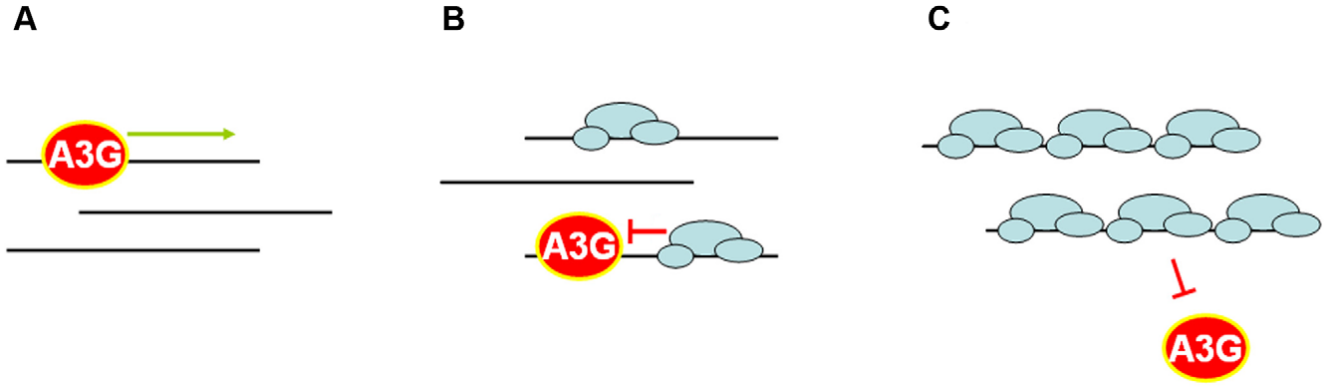
Model of the inhibitory effect of RPA on the deaminase activity of A3G. (a) In the absence of RPA, for example in the cytoplasm, A3G is able to access ssDNA and slide along it (green arrow), catalyzing multiple deaminations. (b) In the presence of intermediate RPA concentrations, only a fraction of ssDNA that is not covered by the RPA is accessible to the deaminase. A3G binds to the available ssDNA regions, but further sliding and/or jumping is hampered by the RPA bound to ssDNA. Processivity of the enzyme is inhibited much stronger than the deamination activity, and the degree of processivity inhibition depends on the extent of coverage of ssDNA by RPA. This scenario likely reflects the situation in the nuclear loci such as the yeast *URA3* gene. (c) High concentrations of RPA completely prevent access of A3G to the ssDNA. It results in 100% inhibition of deamination activity.

We hypothesized that the one important difference between our yeast and *in vitro* systems is the RPA, which is present in live yeast cells but not in the *in vitro* assay. RPA is an eukaryotic ssDNA binding protein [21]. It binds to the ssDNA with high affinity and is involved in replication, DNA repair and recombination. RPA protects ssDNA from damage and prevents secondary structure formation that could influence different DNA transactions. RPA is an abundant nuclear protein that covers ssDNA in the nuclei for a variety of DNA transactions [20], [21]. Since RPA and A3G share a common substrate – ssDNA, it is expected that these two proteins are competing for the nuclear ssDNA pool. Indeed, when we added RPA to the oligonucleotide deaminations reaction, we found that A3G activity is inhibited by both human and yeast RPA (Fig. 6). Moreover, using deaminase assay with gapped DNA substrate we showed that the number of mutant clones with A3G-induced multiple substitutions substantially decreased with the increase in RPA concentration (Fig. 7). This data indicates that RPA suppresses the processive action of A3G. The mechanism of deaminase processivity may include one-dimensional sliding and/or three-dimensional microscopic dissociations and re-associations, called jumping (reviewed with a particular emphasis on APOBEC deaminases in [15]). AID is capable of both sliding and jumping ([18], [19] and references therein). Processivity of A3G may include sliding, jumping and/or inter-segmental transfer [14], [15], [17], [34], [35]. The presence of RPA on the ssDNA will sterically block deaminase sliding, which contributes a lot to deaminase processivity regardless of its ability to jump (for example, see recent model of AID processive action in [19]). Therefore, even deaminase that can jump will have strongly decreased processivity in case its sliding is prevented by the RPA. Additionally, cytosines in the ssDNA regions that are covered with the RPA molecules will not be accessible to the deaminase independent of whether the enzyme moves by sliding, jumping or inter-segmental transfer. Therefore, our data suggest that RPA is a powerful inhibitor of activity and processivity of deaminases in the nuclei (Fig. 9).

Protection of DNA by RPA may take place in the yeast artificial system and probably in the nuclei of vertebrate cells. A3G normally executes its antiviral action in the cytoplasm, where it performs processive deaminations on retroviral cDNAs. RPA is not present in the cytoplasm and therefore does not block the anti-retroviral activity of A3G. Nevertheless, a small but detectable amount of A3G is present in the nuclei of cells endogenously expressing this deaminase [26]. The nuclear level of A3G is also severely increased upon deaminase overproduction. In addition, A3G can accumulate in the nuclei upon some inhibition of proteasome [36]. In agreement with the previous reports, we found by immunofluorescent microscopy that overexpressed A3G in HEK293T cells is localized in the cytoplasm and is found predominantly in the punctuate foci, identified before as P-bodies (Fig. 8a) [27], [28]. We were unable to see A3G in the nuclei of the cells using this method (Fig. 8a). On the other hand, we show by Western blot that A3G is found in the nuclear fraction of these cells (Fig. 8b) (also see [26]). Therefore, it is possible that the nuclear level of A3G is under the limit of detection of fluorescent microscopy. Moreover, treatment with RNAse A during the course of extract fractionation resulted in a significant increase of the A3G nuclear level (Fig. 8b). P-bodies may serve as a storage facility for A3G, preventing it from promiscuous action on non-retroviral cellular DNA [27]. RNAse treatment release A3G from P-bodies [27], which allows it to enter the nucleus by passive diffusion. The presence of cytoplasmic retention signal [25], [37] allows the protein to be predominantly cytoplasmic even when P-bodies are destroyed by RNAse treatment (Fig. 8b). Therefore, multiple mechanisms contribute to the cytoplasmic localization of A3G. Taken together, our data and previous reports suggest that the distribution of A3G between the nucleus and cytoplasm is dynamic and diverse changes in cellular physiology, including those related to pathological conditions, may lead to an accumulation of A3G in the nucleus. Therefore, the genomic DNA of A3G-expressed cells should be protected from the A3G activity. Similarly, when A3G is overexpressed in yeast, it is able to penetrate the nuclei and deaminate genomic DNA by single hits. The results presented in Fig. 3 indicate that the processive action of A3G is suppressed in the yeast genome, whereas the enzyme is robustly processive *in vitro* on the same reporter. In contrast, A3G introduces multiple substitutions in the yeast retroviral-like Ty elements. This is not surprising because Ty reverse transcription takes place in the cytoplasm of yeast cells, where there is no RPA. It has been suggested that, in addition to intracellular compartmentalization, there are additional mechanisms for protection of the genomic DNA from APOBEC3 proteins [5]. Our data suggest that RPA is one of these genomic safeguards.

Similar logic could be applied to the regulation of another deaminase, AID. This enzyme is also processive *in vitro* [13], and its activity is strongly inhibited by RPA or SSB [15], [23]. AID is mutagenic in the yeast system [38] and, similar to A3G, introduces single base substitutions in the *CAN1* reporter gene [10]. AID shuttles between cytoplasm and the nucleus of human cells [39] and represents a potential threat to a genome. Because AID processivity is limited in the Ig genes in SHM and restricted even more in most of the oncogenes that are being mistargeted by this deaminase [32], [33], it is plausible that AID processive activity is also regulated by RPA. Active transcription is a prerequisite for somatic hypermutation, but precise mechanisms that target AID to the Ig genes are unknown. It has been proposed that RPA plays a role in the recruitment of AID to the variable and switch regions of immunoglogulin genes in B-cells [40], [41]. In this model, phosphorylation of AID by the protein kinase A (PKA) allows the AID-RPA interaction, which results in the deamination of the target sites [42]. Precise AID targeting is provided by the recruitment of PKA to the immunoglobulin genes [43]. Recently, it has been proposed that the combined action of transcription factor Spt5 and RPA recruits phosphorylated AID to the Ig loci. ([44] and references therein). However, *in vitro* phosphorylated AID is still inhibited by RPA [23]. Therefore, it is logical to think that AID is recruited to the stalled/paused RNA polymerase II complexes with the aid of Spt5, and this process does not require RPA. There is probably a mechanism for partial RPA exclusion from natural AID ssDNA substrates, which allows AID to target cytosines. Enrichment of RPA in promoters of immunoglobulin genes, that was demonstrated in [44] and used in support of model where RPA attract AID to the target loci, can be explained by recruitment of RPA during the course of DNA repair induced by cytosine deamination, after AID activity is no longer required.

In the genome, RPA binds to ssDNA generated during DNA transactions such as replication, DNA repair and transcription. This, according to our model, prevents deaminase activity on the cytosines located in the ssDNA regions covered by RPA, and processivity, primarily by interfering with enzyme sliding. It is possible that the protection from deaminases by RPA is non-specific by nature and is executed by steric hindrance due to the competition of the RPA and deaminases for the same substrate. The ssDNA that is formed in the course of DNA repair and recombination could also potentially be protected from deaminases by other ssDNA-binding proteins, such as Rad51. A3G works in the cytoplasm, but is also found in the nucleus, where its activity has to be prevented. Different parameters such as transcription activity of particular loci, course of replication and cell cycle progression, as well as tissue- and cell type-specific characteristics, can modify the role of RPA in the prevention of different deaminases access to the genome. Additional studies are required to better understand the mechanism of RPA-based genome protection from the inadvertent deaminases-induced mutagenesis.

## Materials and Methods

### Yeast strains and techniques

Mutant *ura3–4* allele was converted to the wild-type one in the *S. cerevisiae* strain 1B-D770 *ung1::hygB* [45] by transformation with wild type *URA3* DNA obtained by PCR. The resulting strain, named LAN-200 (*MAT*a *ade5-1 lys2*-Tn*5-13 trp1-289 his7-2 leu2-3,112 ung1::hygB*), is suitable for the measurement of the forward mutation rate at the *URA3* locus.

For the mutagenesis experiment, the LAN-200 strain was transformed with pESC-*LEU2* vector and pESC-*LEU2*-hA3G*Sc* expressing plasmid [10]. The human A3G gene in this plasmid is codon-optimized for expression in yeast.

Mutation frequencies were determined by fluctuation analysis as described earlier [46]. Independent yeast transformants were grown in a complete minimal medium without leucine to select for the plasmid. In addition, this media contained galactose and raffinose instead of glucose, to induce A3G expression. Induced cultures were plated undiluted on plates containing 5-FOA to select for *ura3* mutants and with dilution on complete plates to estimate viability. The 5-FOA is converted to the toxic compound by the orotidine 5-phosphate decarboxylase, which is encoded by the *URA3* gene, therefore only *ura3* mutants could grow on the media containing 5-FOA.

To construct the spectra of mutations induced by A3G in yeast, patches of LAN-200 transformants originating from single colonies were replica-plated three times onto fresh medium containing galactose and raffinose but without leucine. Then they were replica-plated onto 5-FOA-containing medium to select for *ura3* mutants. After five days of incubation, independent 5-FOA^R^ colonies were colony-purified on 5-FOA medium. Chromosomal DNA from cells originating from single 5-FOA-resistant colonies was isolated using a Yeast DNA Extraction Kit (Epicentre). Subsequent PCR amplification and sequencing was performed as described previously [38]. Sequences of the primers used for PCR and sequencing are available upon request.

### Cell lines

Human Embryonic Kidney 293T (HEK293T) (Thermo Scientific catalog number HCL4517).

### A3G purification

HEK293T cells were transfected by pcDNA3.1-A3G-Myc-His expression plasmid using polyethileneimine [47]. Purification was done according to protocol [17] with the following modification: buffer containing 500 mM imidazole was used to elute the last three fractions from the resin.

### Oligonucleotide deaminase activity assay

Oligonucleotide deaminase activity assay was performed according to the published method [48]. Briefly, 5′-Cy5-labeled oligonucleotide (5′-Cy5-TTTTTTTTTTTTTTTATCTTTTTTTTTTTACTTTTTTTTTTAAACCCAAATTTTTTTTTTTTTTTTTTTTTTTTTTTTTTTTTTT) was incubated with A3G in the presence of UDG (New England Biolabs). Then abasic sites were converted to strand breaks by heating at high pH. The resulting products were resolved on 16% denaturing PAGE. Gels were scanned using the Typhoon 9410 imaging system (GE Healthcare). Deamination at the CCC site, which represents the A3G hot spot, creates a 47 nucleotide product.

### Electrophoretic Mobility Shift Assay (EMSA)

Electrophoretic Mobility Shift Assay (EMSA) was performed by standard techniques (see [49], for example). The 5′-Cy5 labeled oligonucleotide (5′-Cy5-AAGACCATGACCGCCAGCTCAAGTGTAAGTTACATGCATCTCTACCAGAAGTCAGAGGTTAGATTAGAGAGTATTT) (Integrated DNA Technologies) was incubated with various amounts of A3G in reaction buffer (25 mM Tris-HCl pH 8.0, 50 mM NaCl, 5 mM MgCl_2_ 1 mM DTT, 10% glycerol) for 15 min at 37°C. Then, samples were loaded on 4% acrylamide gel (ratio of acrylamide to bis-acrylamide 75∶1) and run in 0.5× TBE buffer for 3 h. Gels were scanned using the Typhoon 9410 imaging system (GE Healthcare).

### Construction of *pyrF- ung- E.coli* strain

The *ung::tet* marker was transferred to the NR16207 strain (*pyrF::kan*) from the strain BD2328 (*ung::tet*) using P1 phage transduction by Drs. S.G. Kozmin and R. M. Schaaper (NIEHS).

### Construction of gapped DNA substrate

Two plasmids, pRS315-*URA3* OR1 and OR2, which differ in the orientation of the *URA3* gene, were constructed. Circular ssDNA originating from these plasmids was purified from bacteria by standard techniques [50]. Two oligonucleotides are annealed to this ssDNA, first with a free 3′-OH end serving as a primer, the other with a 3′-phosphorylated end blocks DNA synthesis beyond its annealing site (Fig. 4a). The priming oligonucleotide is extended using PfuUltra DNA polymerase. PfuUltra has no strand-displacing activity, therefore, a gapped substrate is formed (Fig. 4b). The gap size is 1110 and 1320 nucleotides when the non-coding and coding strand of *URA3* is in the ssDNA form, respectively. Transformation of the *E.coli pyrF^−^* strain with DNA substrate enables the selection of mutations in the *URA3* gene.

### A3G activity on gapped DNA substrate

Sixty ng of substrate DNA was treated with 100 ng of A3G for 10 min in the 10 µl total volume reactions containing 25 mM Tris-HCl, pH 8.0, 50 mM NaCl, 1 mM DTT, and the products of the reaction were electroporated into the *pyrF- ung- E.coli*. Transformants were selected on LB plates with ampicilin, streaked on the same type of plates and then replica-plated on Vogel-Bonner media plates, with and without uracil, to select for *ura3* mutants [51]. Dependent on the experiment, 4 to 8 percent of the clones selected on the LB+Amp plates exhibited a mutant phenotype, which is indicative of the processive mechanism of enzyme action according to Poisson statistics (*p*<10^−7^; see Table S1) [13], [18], [19]. The *URA3* gene from *ura^−^* clones was sequenced. Recombinant RPA was added to the deamination reactions where applicable.

### Mutation spectra construction and analysis

DNA Star 8 (Lasergene) software was used for sequence analyses. The χ^2^ test was used to test the hypothesis that A3G works processively in gapped substrate assay (Table S1) and to compare the frequencies of clones with multiple mutations (Figure 7). The same test was used to confirm that APOBEC3G is non-processive *in vivo* in yeast. Calculations were done using the COLLAPSE [52] and STATISTICA programs [52]. The Pearson linear correlation coefficient was used to compare spectra. Calculations were done using the program STATISTICA [52].

### Immunocytochemistry and fluorescent microscopy

HEK293T cells transiently transfected with pcDNA3.1-A3G-Myc-His plasmid or mock control cells were fixed with methanol-acetic acid mixture (3∶1). All procedures were performed at room temperature. Mouse anti-His_6_-tag antibodies (GenScript, USA) and goat-anti-mouse Cy3-conjugated antibodies (ThermoScientific, USA) were used for A3G detection. Cells were mounted in Vectashield mounting medium with DAPI (Vector Laboratories, Burlingame, CA). Images were captured with a Zeiss Axiovert 200 M microscope (Carl Zeiss, Thornwood, NY) equipped with an ORCA-ER digital camera (Hamamatsu, Hamamatsu City, JP) and processed using OpenLab software (Improvision, Boston, MA).

### Nuclear-cytoplasmic fractionation and Western-blot analysis

HEK293T cells transfected with the pcDNA3.1-A3G-Myc-His plasmid or mock control cells were collected from 10-cm culture dishes 26 hours after transfection (about 100% final confluency), washed once with PBS and resuspended in 300 µl of PBS buffer containing 1% Triton X-100, 2 mM DTT, protease inhibitors cocktail set IV (100× dilution, Calbiochem), 5 mM PMSF, and incubated for 5 min on ice. Lyzates then were split into two aliquots, and 10 µg of RNAse A (Qiagen) was added to one of the aliquots. After incubation for 30 min at 37°C the lyzates were layered on top of 1 ml of 30% sucrose/PBS/DTT buffer. Samples were spun at 800 g for 10 min at 4°C and supernatant (cytoplasmic fraction) was saved. Nuclear pellet was washed once with PBS and resuspended in 1.3 ml of RIPA buffer containing 2 mM DTT, protease inhibitors cocktail set IV (100× dilution, Calbiochem), 5 mM PMSF. After 10 min incubation on ice, lyzates were spun at 15000 g for 10 min at 4°C, and supernatant (nuclear fraction) was saved. Both nuclear and cytoplasmic fractions were used for Western blotting. Mouse Anti-His_6_-tag antibodies were used to detect A3G-Myc-His and mouse anti-α-tubulin antibodies (both from Genscript) were used to confirm efficient nuclear-cytoplasmic fractionation.

## Supporting Information

Table S1 **Analysis of mutations introduced by APOBEC3G into gapped DNA substrate** ***in vitro*** **.** Thirty-nine mutant clones obtained in one experiment are shown. Numbers in “Substitutions” column indicate nucleotide positions in the *URA3* ORF. Track size is defined as the distance (bp) between first and last substitutions. If APOBEC3G works in distributive fashion, then the frequencies of clones with certain number of substitutions should follow the Poisson distribution. We have found, on the contrary, that the observed distribution is strikingly different from the expected Poisson distribution (*p*<10^−7^ according to the χ^2^ test for the data of the experiment presented in the table), confirming that tracts of mutations found result from processive action of APOBEC3G.
(DOC)
