# Supplementary material for: Replication Protein A (RPA) Hampers the Processive Action of APOBEC3G Cytosine Deaminase on Single-Stranded DNA
Source: PLoS One. 2011 Sep 15;6(9):e24848. doi: 10.1371/journal.pone.0024848 (PMC3174200; doi:10.1371/journal.pone.0024848)
Supplement: Table S1 — Analysis of mutations introduced by APOBEC3G into gapped DNA substrate in vitro . Thirty-nine mutant clones obtained in one experiment are shown. Numbers in “Substitutions” column indicate nucleotide positions in the URA3 ORF. Track size is defined as the distance (bp) between first and last substitutions. If APOBEC3G works in distributive fashion, then the frequencies of clones with certain number of substitutions should follow the Poisson distribution. We have found, on the contrary, that the observed distribution is strikingly different from the expected Poisson distribution (p<10−7 according to the χ2 test for the data of the experiment presented in the table), confirming that tracts of mutations found result from processive action of APOBEC3G. (DOC) [file pone.0024848.s001.doc]

Supporting Table 1. Analysis of mutations introduced by APOBEC3G into gapped DNA substrate *in vitro*.

Thirty-nine mutant clones obtained in one experiment are shown. Numbers in “Substitutions” column indicate nucleotide positions in the *URA3* ORF. Track size is defined as the distance (bp) between first and last substitutions. If APOBEC3G works in distributive fashion, then the frequencies of clones with certain number of substitutions should follow the Poisson distribution. We have found, on the contrary, that the observed distribution is strikingly different from the expected Poisson distribution (*p*<10^-7^ according to the χ^2^ test for the data of the experiment presented in the table), confirming that tracts of mutations found result from processive action of APOBEC3G.

| clone # | # of sbst | Track size | density (substitutions per 100 bp) | Substitutions |
| --- | --- | --- | --- | --- |
| 1 | 8 | 60 | 13.3 | G720A G725A G726A G735A G741A G767A G768A G780A |
| 2 | 11 | 660 | 1.7 | G108A G321A G464A G468A G564A G568A G577A G639A G660A G767A G768A |
| 3 | 17 | 655 | 2.6 | G135A G136A G213A G234A G292A G321A G546A G568A G612A G627A G679A G700A G725A G741A G767A G768A G790A |
| 4 | 15 | 582 | 2.6 | G186A G321A G367A G468A G484A G498A G564A G565A G580A G639A G660A G725A G741A G767A G768A |
| 5 | 8 | 386 | 2.1 | G382A G468A G541A G564A G679A G745A G767A G768A |
| 6 | 31 | 672 | 4.6 | G108A G126A G186A G213A G321A G322A G344A G375A G468A G484A G498A G529A G564A G565A G568A G580A G590A G607A G612A G627A G639A G660A G679A G700A G706A G720A G725A G741A G767A G768A G780A |
| 7 | 16 | 459 | 3.5 | G321A G382A G468A G484A G498A G564A G577A G590A G612A G660A G700A G720A G725A G767A G768A G780A |
| 8 | 17 | 660 | 2.6 | G108A G126A G213A G321A G344A G375A G468A G498A G564A G568A G607A G639A G660A G720A G735A G767A G768A |
| 9 | 17 | 641 | 2.7 | G126A G186A G321A G375A G394A G408A G468A G484A G564A G612A G627A G639A G679A G720A G725A G741A G767A |
| 10 | 15 | 660 | 2.3 | G108A G321A G322A G344A G367A G468A G564A G639A G669A G679A G720A G725A G741A G767A G768A |
| 11 | 18 | 459 | 3.9 | G321A G344A G375A G468A G484A G564A G612A G627A G660A G679A G700A G706A G720A G725A G741A G767A G768A G780A |
| 12 | 29 | 642 | 4.5 | G126A G210A G213A G292A G322A G344A G375A G468A G484A G498A G541A G564A G568A G580A G590A G607A G612A G627A G639A G660A G679A G700A G720A G725A G726A G735A G741A G767A G768A |
| 13 | 22 | 614 | 3.6 | G154A G186A G292A G321A G468A G484A G498A G564A G590A G607A G612A G613A G619A G639A G660A G679A G700A G720A G721A G725A G767A G768A |
| 14 | 9 | 270 | 3.3 | G498A G564A G607A G627A G639A G679A G726A G767A G768A |
| 15 | 20 | 582 | 3.4 | G186A G210A G213A G321A G344A G375A G426A G468A G498A G564A G586A G612A G627A G628A G660A G700A G725A G741A G767A G768A |
| 16 | 24 | 660 | 3.6 | G108A G135A G213A G321A G344A G375A G468A G498A G541A G564A G607A G612A G627A G639A G660A G669A G679A G700A G720A G725A G741A G742A G767A G768A |
| 17 | 13 | 757 | 1.7 | G10A G154A G210A G321A G344A G375A G468A G484A G564A G607A G612A G700A G767A |
| 18 | 10 | 582 | 1.7 | G186A G321A G344A G564A G568A G607A G720A G741A G767A G768A |
| 19 | 16 | 632 | 2.5 | G135A G321A G367A G394A G468A G498A G541A G564A G568A G612A G679A G700A G720A G725A G741A G767A |
| 20 | 17 | 660 | 2.6 | G108A G321A G344A G375A G468A G498A G564A G565A G568A G580A G607A G660A G700A G725A G741A G767A G768A |
| 21 | 16 | 642 | 2.5 | G126A G186A G234A G321A G468A G484A G498A G607A G612A G660A G679A G720A G725A G741A G767A G768A |
| 22 | 18 | 582 | 3.1 | G186A G321A G344A G345A G349A G375A G468A G484A G564A G568A G612A G627A G679A G700A G720A G725A G767A G768A |
| 23 |  |  |  | G767A |
| 24 | 10 | 633 | 1.6 | G135A G321A G468A G498A G564A G612A G679A G700A G767A G768A |
| 25 | 12 | 747 | 1.6 | G21A G108A G321A G344A G564A G568A G607A G627A G725A G741A G767A G768A |
| 26 | 18 | 660 | 2.7 | G108A G126A G213A G292A G321A G344A G375A G468A G498A G564A G568A G590A G607A G639A G720A G742A G767A G768A |
| 27 | 9 | 659 | 1.4 | G108A G321A G468A G498A G564A G639A G720A G726A G767A |
| 28 | 14 | 762 | 1.8 | G6A G126A G321A G375A G468A G564A G580A G612A G679A G720A G735A G741A G767A G768A |
| 29 | 20 | 660 | 3.0 | G108A G135A G186A G321A G322A G344A G375A G382A G468A G484A G498A G612A G627A G639A G700A G720A G725A G741A G767A G768A |
| 30 | 4 | 60 | 6.7 | G720A G767A G768A G780A |
| 31 | 21 | 659 | 3.2 | G108A G135A G186A G213A G321A G344A G357A G372A G375A G468A G484A G498A G564A G565A G568A G612A G700A G706A G720A G741A G767A |
| 32 | 10 | 555 | 1.8 | G213A G321A G468A G541A G564A G680A G725A G741A G767A G768A |
| 33 | 7 | 447 | 1.6 | G321A G375A G468A G564A G660A G767A G768A |
| 34 | 10 | 659 | 1.5 | G108A G213A G321A G468A G485A G498A G612A G720A G725A G767A |
| 35 | 16 | 581 | 2.8 | G186A G321A G344A G375A G394A G468A G498A G564A G568A G612A G627A G660A G720A G721A G725A G767A |
| 36 | 16 | 582 | 2.7 | G186A G321A G375A G468A G484A G498A G564A G607A G612A G627A G660A G700A G706A G725A G767A G768A |
| 37 | 20 | 594 | 3.4 | G186A G210A G322A G344A G376A G468A G484A G485A G498A G564A G568A G580A G627A G679A G700A G706A G725A G741A G767A G780A |
| 38 | 13 | 655 | 2.0 | G135A G186A G213A G321A G375A G468A G564A G700A G725A G763A G767A G768A G790A |
| 39 | 11 | 656 | 1.7 | G112A G321A G468A G498A G564A G568A G577A G607A G720A G767A G768A |
| average | 15.2 | 581 | 3.0 |  |
| MIN | 4 | 60 | 1.4 |  |
| MAX | 31 | 762 | 13.3 |  |
